# A systematic review of explainable artificial intelligence methods for speech-based cognitive decline detection

**DOI:** 10.1038/s41746-025-02105-z

**Published:** 2025-11-26

**Authors:** Ravi Shankar, Ziyu Goh, Fiona Devi, Qian Xu

**Affiliations:** 1 Clinical Research & Innovation Office, Tan Tock Seng Hospital, National Healthcare Group, Singapore, 308433 Singapore; 2 Yong Loo Lin School of Medicine, National University of Singapore, Singapore, 117597 Singapore; 3 Medical Affairs – Research Innovation & Enterprise, Alexandra Hospital, National University Health System, Singapore, 159964 Singapore; 4 School of Civil, Aerospace and Design Engineering, University of Bristol, Bristol, BS8 1TH UK

**Keywords:** Computational biology and bioinformatics, Health care, Mathematics and computing, Medical research

## Abstract

Artificial intelligence models analyzing speech show remarkable promise for identifying cognitive decline, achieving performance comparable to clinical assessments. However, their “black box” nature poses significant barriers to clinical adoption, as healthcare professionals require transparent decision-making processes. This challenge is compounded by regulatory requirements, including GDPR mandates for explainability and medical device regulations emphasizing AI transparency. Following PRISMA guidelines, we systematically reviewed explainable AI (XAI) techniques for speech-based detection of Alzheimer’s disease and mild cognitive impairment across six databases through May 2025. From 2077 records, 13 studies met the inclusion criteria, employing XAI methods including SHAP, LIME, attention mechanisms, and novel approaches across machine learning architectures. Models achieved AUC values of 0.76-0.94, consistently identifying acoustic markers (pause patterns, speech rate) and linguistic features (vocabulary diversity, pronoun usage). While XAI techniques demonstrate promise for clinical interpretability, significant gaps remain in stakeholder engagement, real-world validation, and standardized evaluation frameworks.

## Introduction

As of 2019, an estimated 57 million individuals worldwide were living with dementia, the majority of whom had Alzheimer’s disease and other dementias (ADODs), and this number is projected to rise to 153 million by 2050^1^. This reflects a dramatic global increase in dementia prevalence, largely driven by aging populations, particularly in low- and middle-income countries^2^. Early detection of cognitive decline is crucial for timely intervention, treatment planning, and support for patients and caregivers. However, current diagnostic approaches face significant limitations^3^. Neuropsychological assessments require specialized training and can be time-consuming, while neuroimaging techniques such as PET scans and MRI are expensive and not universally accessible^4^. These constraints have driven the search for more accessible, cost-effective screening methods that can be deployed at scale.

Speech and language changes often manifest as early indicators of cognitive decline, sometimes preceding other clinical symptoms by several years^5^. These changes encompass multiple dimensions: reduced lexical diversity, increased use of pronouns and filler words, simplified syntactic structures, altered speech fluency, and changes in acoustic properties such as pause patterns and articulation rate^6,7^. The multifaceted nature of these speech biomarkers makes them particularly suitable for AI-based analysis, which can capture subtle patterns across multiple features simultaneously.

Advances in natural language processing (NLP) and machine learning (ML) have enabled the detection of these subtle speech markers with accuracies exceeding 90% in distinguishing cognitively normal individuals from those with dementia^8,9^. These AI models analyze a comprehensive range of features, including acoustic characteristics (such as pitch variability and speech rate), linguistic markers (vocabulary richness, grammatical complexity), and semantic content (coherence, information density)^10^.

Despite their impressive performance, the clinical adoption of AI-based cognitive assessment tools remains limited. A primary barrier is the “black box” nature of many AI models, particularly deep learning architectures, which provide little insight into their decision-making processes^11^. This lack of transparency creates several critical challenges. Healthcare professionals need to understand the reasoning behind AI predictions to effectively integrate them into diagnostic and treatment decisions. Without clear explanations, clinicians may be reluctant to rely on AI recommendations, particularly in high-stakes diagnostic scenarios. Medical device regulations increasingly require transparency and interpretability in AI systems^12^. The European Union’s Medical Device Regulation (MDR) and the FDA’s guidelines on AI/ML-based medical devices emphasize the need for explainable decision-making processes^13^. Additionally, the General Data Protection Regulation (GDPR) explicitly mandates explainability for automated decision-making systems, creating both legal and clinical imperatives for transparent AI implementations in healthcare settings.

When AI models make incorrect predictions, the lack of interpretability makes it difficult to identify the source of errors or to determine when the model might be unreliable for specific patient populations or clinical contexts. Clinicians need to explain diagnostic decisions to patients and their families. Opaque AI predictions complicate this communication and may undermine patient trust in the diagnostic process.

Explainable AI (XAI) methods aim to address these challenges by making AI models more interpretable and transparent^14^. XAI encompasses a diverse set of techniques designed to provide insights into model behavior, feature importance, and decision rationale. In the context of cognitive decline detection, XAI can serve multiple purposes. Feature attribution identifies which speech features (e.g., pause frequency, word choice, acoustic properties) most strongly influence the model’s predictions, allowing clinicians to understand the linguistic and acoustic markers driving the assessment^15^. Clinical alignment maps AI model behavior to established clinical knowledge about speech changes in dementia, validating that the model is learning clinically relevant patterns rather than spurious correlations. Individual explanations provide patient-specific explanations that highlight the particular speech characteristics contributing to their risk assessment, enabling personalized clinical insights. Quality assurance enables clinicians to verify that model predictions are based on appropriate features and to identify potential biases or limitations in the model’s reasoning. For this review, we adopted a comprehensive definition of XAI encompassing not only post-hoc explanation methods like SHAP and LIME, but also attention mechanisms in neural networks, rule-based interpretable models, and intrinsically transparent algorithms that provide inherent interpretability.

While individual studies have begun incorporating XAI techniques into speech-based cognitive assessment systems, the field lacks a comprehensive synthesis of these approaches. Key questions remain unanswered regarding which XAI techniques are most effective for different types of speech analysis models and clinical applications, how well current XAI implementations align with clinical needs and workflows, what evidence exists for the clinical utility and impact of XAI-enhanced cognitive assessment tools, and what technical and practical challenges exist in implementing XAI for speech-based cognitive screening.

This systematic review aims to address these knowledge gaps by providing a comprehensive overview of the speech datasets, AI architectures, and XAI techniques currently used for cognitive decline detection. While previous systematic reviews have examined AI applications in speech-based dementia detection generally^8,16^, this review is the first to specifically focus on explainable AI implementations. Unlike prior work that primarily assessed predictive performance, our review uniquely evaluates interpretability methods, clinical translation efforts, and stakeholder engagement, thereby addressing the critical gap between AI development and clinical adoption that has emerged as regulatory and clinical demands for transparency have intensified. We analyze the technical implementation and evaluation of XAI methods across studies, including their strengths and limitations. We examine the clinical interpretability and translation of XAI outputs for cognitive assessment, including stakeholder engagement and validation approaches. We identify open challenges, methodological limitations, and future directions for advancing XAI in this domain. Finally, we develop recommendations for researchers and clinicians on best practices for implementing and evaluating XAI in speech-based cognitive assessment.

By critically appraising the latest research through an XAI lens, this review provides actionable insights for developing more interpretable, trustworthy, and clinically useful AI screening tools for early dementia detection. The synthesis of technical approaches, clinical applications, and implementation challenges offers a roadmap for advancing the field toward practical, deployable solutions that can enhance clinical decision-making while maintaining transparency and trust.

## Results

### Study selection

**Fig. 1:**
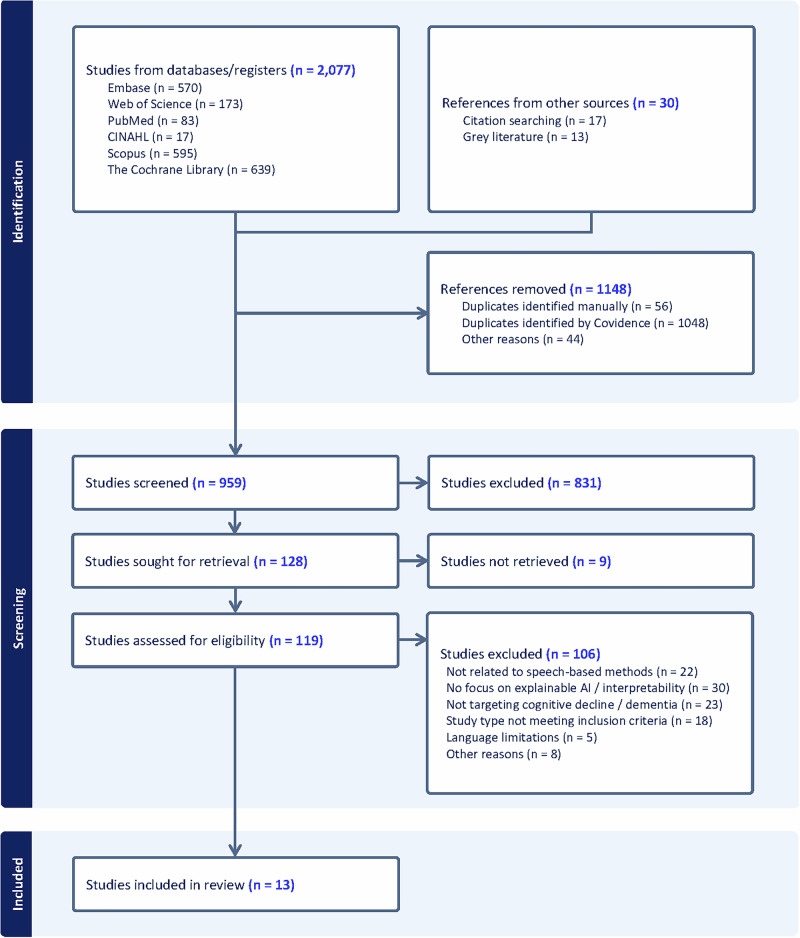
PRISMA flow diagram illustrating the study selection process. The systematic review identified 2077 initial studies from database searches and 30 additional references from other sources. After removing 1148 duplicates, 959 studies were screened. Full-text review of 128 articles resulted in 13 studies meeting all inclusion criteria for final analysis.

The systematic search across six databases yielded 2077 records. After removing 1118 duplicates, 959 unique records underwent title and abstract screening. Of these, 831 were excluded as clearly not meeting the inclusion criteria, leaving 128 records for full-text assessment. Following detailed full-text review, 115 studies were excluded for the following reasons: no explainable AI component implemented (*n* = 30, including 18 studies using only traditional machine learning methods and 12 using statistical analyses without XAI), not focused on speech-based analysis (*n* = 22), not targeting cognitive decline/dementia (*n* = 23), wrong study type including reviews, protocols, and abstracts (*n* = 18), language limitations (*n* = 5), study non-retrieval (*n* = 9), and other reasons (*n* = 8). Ultimately, 13 studies met all inclusion criteria and were included in the systematic review. The PRISMA flow diagram illustrates the complete study selection process (Fig. 1).

### Workflow overview

**Fig. 2:**
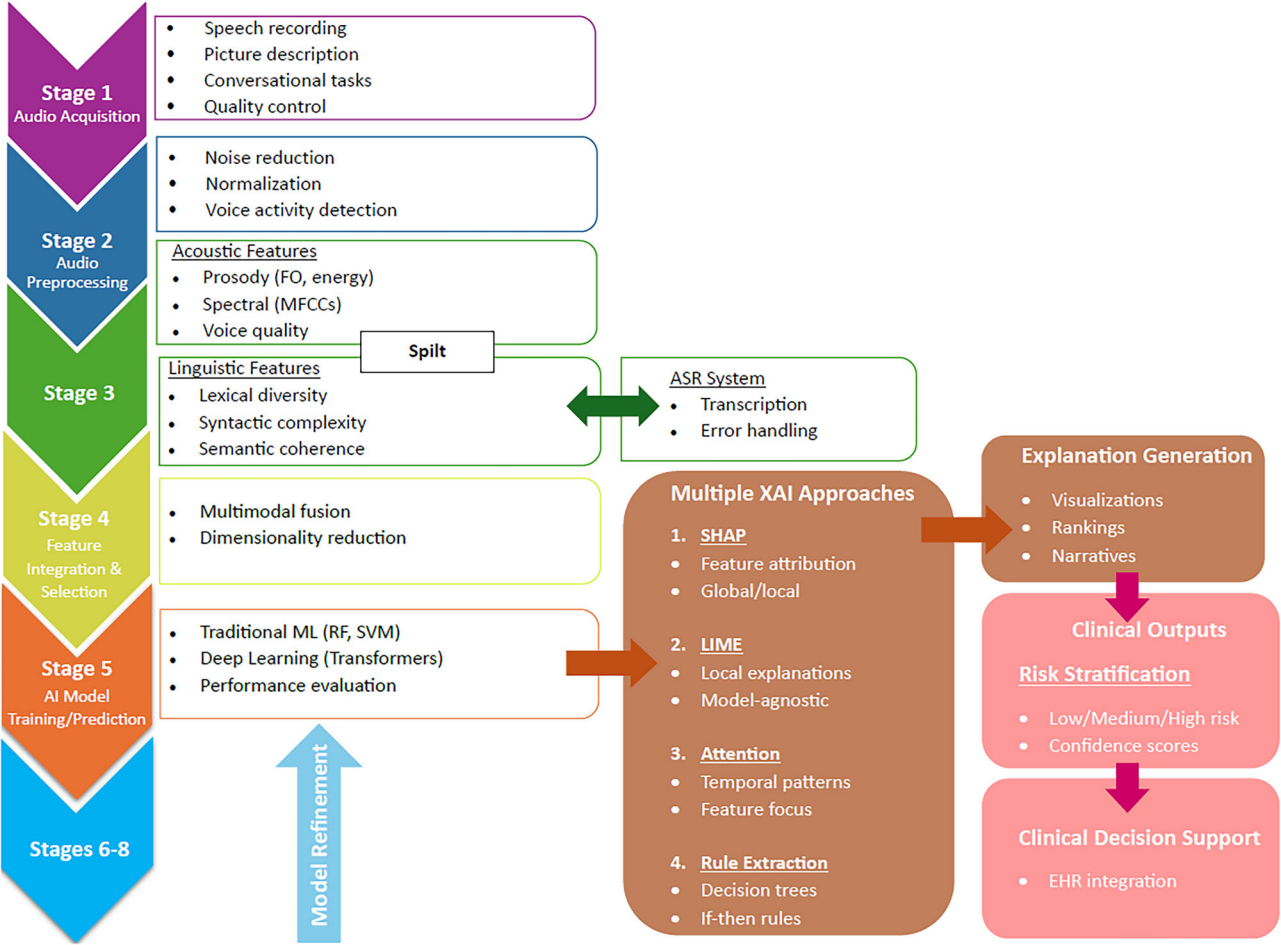
Typical workflow for XAI-enhanced speech-based cognitive assessment. Standard pipeline showing the progression from audio acquisition through feature extraction, model training, explanation generation, and clinical interpretation. The workflow encompasses speech task collection (picture description, conversation), preprocessing steps (noise reduction, normalization), feature extraction (acoustic and linguistic), AI model prediction, XAI method application (SHAP, LIME, attention mechanisms), and clinical decision support integration.

Figure 2. presents a typical workflow for XAI-enhanced speech-based cognitive assessment, illustrating the standard pipeline from audio acquisition through feature extraction, model training, explanation generation, and clinical interpretation.

### Study Characteristics

The included studies were published between 2021 and 2025, with a notable increase in publications from 2023 onward, including one study in 2021, one in 2022, three in 2023, four in 2024, and four in 2025. This temporal distribution reflects the recent emergence of XAI as a priority in healthcare AI applications. Studies originated from diverse geographical regions, with five from Europe (Spain, Greece, UK), four from Asia (Hong Kong, China), three from North America (USA, Canada), and one international collaboration. All included studies were experimental in design, with primary objectives including development of interpretable models for AD/MCI detection (*n* = 8), comparison of XAI techniques for speech analysis (*n* = 3), and clinical validation of XAI-enhanced systems (*n* = 2). Sample sizes varied considerably, ranging from 42 to 758 participants with a median of 162 and interquartile range of 156-291. The total number of unique participants across all studies was approximately 2,800, though some studies used overlapping public datasets.

### Population characteristics

The combined participant demographics across studies revealed mean ages ranging from 63.1 to 85 years, with most reporting participants in their 70 s. Age matching between cognitive groups was attempted in nine studies, though four reported significant age differences between controls and patients. Overall, there was a slight female predominance at 58% female and 42% male, consistent with dementia epidemiology, though individual studies showed considerable variation ranging from 33% to 67% female. Only five studies reported education levels, with means ranging from 12.1 to 15.3 years, representing a significant gap in understanding potential confounders. Language and cultural diversity were limited, with ten studies using English, three multilingual studies including Cantonese, Italian, and Spanish, and most studies lacking ethnic/racial diversity reporting.

Across all studies, participants were distributed as follows: cognitively normal controls comprised 1245 participants (44.5%), mild cognitive impairment included 178 participants (6.4%), Alzheimer’s disease encompassed 821 participants (29.3%), other or mixed dementia included 352 participants (12.6%), subjective cognitive complaints comprised 9 participants (0.3%), and 195 participants (6.9%) were unspecified. Diagnostic criteria varied across studies, with eight using clinical diagnosis by experts, four using standardized criteria such as DSM-5 or NIA-AA, and one study using cognitive test scores only.

Current dataset sizes and diversity constrain practical usability claims. The median sample of 162 participants (IQR: 156-291) falls below thresholds for robust machine learning, particularly for deep learning requiring thousands of samples. Six studies using the same 156-participant ADReSS subset limit independent validation. Population homogeneity further restricts generalizability: ten studies used exclusively English speakers, most lacked ethnic/racial diversity reporting, and only five reported education levels. Vocabulary-based features may not generalize across educational levels or languages, and pause patterns may differ across cultural conversational norms. Clinical deployment requires validation in larger diverse cohorts (*n* > 1000), prospective real-world settings, multiple languages, and populations with comorbidities reflecting clinical heterogeneity. Current evidence supports proof-of-concept, not practical deployment.

### Speech data characteristics

Studies employed diverse speech elicitation tasks. Picture description was used in nine studies, with seven using the Cookie Theft picture from the Boston Diagnostic Aphasia Examination and two using other pictures, typically requiring 1–3 min. Sequential narrative tasks were employed in two studies, including the Rabbit Story with a 15-image sequence in one study and custom multi-image tasks in another. Conversational speech was utilized in three studies through semi-structured interviews, free conversation, and topic-prompted discussion. Other tasks used in four studies included story recall, verbal fluency tests, reading tasks, and memory description.

Public datasets were commonly used, with ADReSS/ADReSSo (Alzheimer’s Dementia Recognition through Spontaneous Speech) employed in six studies, DementiaBank/Pitt Corpus in four studies, and the Lu Corpus in one study. Custom datasets included CU-MARVEL-RABBIT with 758 participants in one study and local clinical collections in four studies. Recording conditions were predominantly controlled laboratory or clinic settings in ten studies, home or telephone-based in two studies, and mixed settings in one study.

### Speech processing and feature extraction

Common preprocessing steps included noise reduction in eight studies, volume normalization in seven studies, voice activity detection in six studies, and sampling rate standardization, typically to 16 kHz, in nine studies. Studies extracted diverse feature sets across multiple categories. Acoustic features were used in twelve studies and included prosodic features such as pitch (F0) statistics, energy, and speaking rate; spectral features including MFCCs, formants, and spectral centroid; voice quality measures like jitter, shimmer, and harmonic-to-noise ratio; and temporal features encompassing pause patterns, silence ratios, and phonation time. Linguistic features were employed in nine studies, covering lexical aspects like vocabulary diversity, word frequency, and type-token ratio; syntactic measures including parse tree depth, POS tags, and grammatical complexity; semantic features such as word embeddings, coherence measures, and information content; and psycholinguistic categories from LIWC and concreteness ratings. Combined approaches were used in seven studies, integrating acoustic and linguistic feature fusion, multimodal embeddings, and end-to-end deep features.

For transcript-based analysis, various automatic speech recognition systems were employed. Google Cloud Speech-to-Text was used in two studies, OpenAI Whisper in four studies, and other ASR systems in three studies, while three studies relied on manual transcription only. Notably, only three studies reported word error rates, which ranged from 31% to 34%.

### AI model architectures

Traditional machine learning approaches were used in eight studies. Support Vector Machines (SVM) were employed in four studies using linear and RBF kernels, achieving AUC values of 0.76–0.89. Random Forest classifiers were the most common traditional approach, used in five studies and often showing the best performance among traditional models with accuracies of 75–89%. Other traditional models included Logistic Regression in four studies, XGBoost in three studies, and Naïve Bayes in two studies.

Deep learning approaches were utilized in five studies. Transformer-based models included BERT variants in three studies and custom transformers in two studies, generally achieving superior performance with AUC values of 0.84–0.94. Other deep architectures included CNNs in one study, LSTMs in one study, and attention networks in two studies. Performance metrics varied by task and dataset, with binary classification of AD versus control achieving AUC values of 0.76–0.94, multi-class classification including MCI showing accuracies of 71–96%, MMSE prediction demonstrating MAE of 3.7–4.7 points, and the best overall performance coming from multimodal transformer approaches (Table 1).

**Table 1:** Technical Implementation and Evaluation of XAI Methods

Study	XAI method(s) and implementation	Feature types and base models	Performance metrics	Evaluation framework and stability assessment	Quantitative metrics	Datasets used
Heitz et al. (2024)^17^	- SHAP - Applied to Random Forest - Mean absolute SHAP value for importance	- Lexical - Syntactic - Semantic Models: - BERT - Random Forest	AUROC: - Manual BERT: 0.899 - Manual RF: 0.888 - ASR BERT: 0.837 - ASR RF: 0.865	- Cross-validation - Feature stability measurement - Most stable: avg_word_length, mattr - Most unstable: flesch_kincaid - Balanced dataset validation	- Feature stability (sj metric) - SHAP value distribution	ADReSS Challenge Dataset
Ilias & Askounis (2022)^18^	- LIME - 5000 samples - Model-agnostic approach	- Lexical - Syntactic - Semantic Models: - BERT variants - BioBERT - Siamese networks	- Accuracy: 87.50% - F1-score: 86.73%	- Statistical significance testing - Feature correlation analysis - POS tag stability analysis	- Point-biserial correlation - Jaccard’s index - 5-fold cross-validation	ADReSS Dataset
de Arriba-Pérez et al. (2024)^27^	- Meta-transformer wrapper - Tree algorithm-based - Component-based analysis	- Content-independent - High-level reasoning - Context-dependent Models: - Random Forest - Decision Tree - Naive Bayes - LLM (ChatGPT)	- Accuracy: 98.47% - Precision: 98.49%	- Component-based analysis - Feature selection metrics - Context-independent features	- Mean Decrease in Impurity - Pearson correlation - 10-fold cross-validation	Celia web application dataset
Ambrosini et al. (2024)^28^	- SHAP - Feature attribution method - Feature importance ranking	- Voice periodicity - Spectral - Syllabic Models: - SVM - CatBoost - Logistic Regression	- Italian: 80-86% - Spanish: Lower performance	- Multi-language validation - Cross-lingual stability - Language-specific features	- Feature attribution scores - Cross-lingual metrics - Multi-center validation	Custom dataset
Tang et al. (2023)^19^	- SHAP - Open-source Python package - Global feature importance	- Lexical - Syntactic - Semantic Models: - SVM - MLP - AdaBoost - Ensemble	- Accuracy: 89.58% - AUC: 0.9531	- Global-local interpretation - Feature ranking - ASR stability analysis - Feature elimination	- SHAP values - Feature importance scores	ADReSS-IS2020 dataset
Chandler et al. (2023)^30^	- Feature attribution methods - Decision Tree extraction - Statistical testing	- Lexeme-level - Syntactic - Semantic Models: - Random Forest	- Overall: 75% - F1-scores: 0.72-0.77	- Statistical testing - Clinical correlation - Temporal stability	- F-statistic - Univariate selection - Clinical validation	Custom dataset
Iqbal et al. (2024)^24^	- LIME + SHAP - Feature importance extraction - Statistical testing	- Lexical - Syntactic - POS tags Models: - Random Forest	- Accuracy: 80% - F1-score: 79-81%	- LIME-SHAP comparison - Statistical validation - POS tag stability	- Confidence metrics - Feature importance - Random search	DementiaBank (ADReSSo challenge dataset)
Han et al. (2025)^25^	- SHAP (Tree-based) - Counterfactual generation via LLM - Chain-of-thought prompting	- TF-IDF features - Pause features Model: - XGBoost	- Sensitivity: 4% → 42% - F1-score: 4% → 35%	- Feature analysis before/after generation - Euclidean distance metrics	- SHAP values - Feature elimination - Distance metrics	Pitt corpus from DementiaBank
Oiza-Zapata & Gallardo-Antolín (2025)^20^	- SHAP - Mutual Information - Dual use for selection & interpretation	- eGeMAPS (88 features) Models: - SVM - Random Forest - XGBoost	- Accuracy: 75.00% - AUC: 0.76 - CUI: 0.5643	- Clinical Utility Index - ~70% computation reduction - Systematic evaluation	- SHAP importance - MI scores - Clinical utility	ADReSS Dataset
Jang et al. (2021)^29^	- Feature importance analysis - T-statistics from LR coefficients - Odds ratio interpretation	- Acoustic (MFCCs) - Linguistic - Eye-tracking Models: - Logistic Regression - Random Forest - Gaussian Naïve Bayes	- AUC: 0.83 ± 0.01 - Task fusion performance	- 10-fold cross-validation - Feature ranking - Multi-modal analysis	- T-statistics - Odds ratios - Confidence intervals	Custom dataset
Li et al. (2025)^26^	- SHAP - Attention mechanisms - Correlation analysis	- Acoustic - Linguistic - Topic modeling (DTM) Models: - SVM - TITAN (custom)	- Accuracy: 71.0% - AUC: 0.8120 - F1: 0.7238	- Spearman correlation - Cross-modal consistency - Temporal analysis	- SHAP beeswarm plots - Attention weights - R² = 0.3876	CU-MARVEL-RABBIT Corpus, ADReSS
Lima et al. (2025)^21^	- SHAP (TreeExplainer) - Feature importance - Risk stratification	- NLP features (100) - eGeMAPS - GPT embeddings Models: - Random Forest - XGBoost - DNN	- Accuracy: 76.5% - AUC: 0.857 - MAE: 3.7 (MMSE)	- 10-fold cross-validation - External validation - Demographic parity	- SHAP values - Feature rankings - Risk categories	ADReSSo, Lu Corpus, Pilot study
Ntampakis et al. (2025)^22^	- Attention visualization - RAG-based explanations - Literature grounding	- Acoustic (47) - Wav2Vec2 embeddings - DeBERTa embeddings Model: - Multimodal Transformer	- Accuracy: 95.77% - F1: 0.9576	- Medical professional evaluation - Interpretability: 3.96/5 - Clinical relevance: 3.85/5	- Attention maps - Explanation quality scores - Clinical utility: 3.70/5	IS2021 ADReSSo Challenge Dataset

*AD* Alzheimer’s disease, *ADReSS* Alzheimer’s dementia recognition through spontaneous speech, *ASR* automatic speech recognition, *AUC* area under the curve, AUROC area under the receiver operating characteristic curve, *BERT* bidirectional encoder representations from transformers, *BioBERT* Biomedical BERT, *CNN* convolutional neural network, *CUI* Clinical Utility Index, *CV* cross-validation, *DeBERTa* decoding-enhanced BERT with disentangled Attention, *DTM* Dynamic Topic Model, *eGeMAPS* extended Geneva Minimalistic Acoustic Parameter Set, F1 = F1-score, *GPT* generative pre-trained transformer, *LIME* local interpretable model-agnostic explanations, *LIWC* linguistic inquiry and word count, *LLM* large language model, *LR* logistic regression, *LSTM* long short-term memory, *MAE* mean absolute error, *MCI* mild cognitive impairment, *MFCC* Mel-frequency Cepstral coefficients, *MI* mutual information, *MLP* multi-layer perceptron, *MMSE* mini-mental state examination, *NLP* natural language processing, *POS* part-of-speech, *QUADAS-2* Quality Assessment of Diagnostic Accuracy Studies-2, *RAG* retrieval-augmented generation, *RF* Random Forest, *RNN* recurrent neural network, *SHAP* SHapley Additive exPlanations, *SVM* support vector machine, *TF-IDF* term frequency-inverse document frequency, *TITAN* text-image temporal alignment network, *Wav2Vec2* wave-to-vector version 2, *XAI* explainable artificial intelligence, *XGBoost* eXtreme gradient boosting

Dataset overlap existed across the included studies, with six studies utilizing the ADReSS/ADReSSo dataset^17–22^, most analyzing identical 156-participant subsets^23^ from the challenge dataset. Two studies used the DementiaBank/Pitt Corpus^24,25^ with different participant cohorts. Li et al.^26^ employed both ADReSS and their novel CU-MARVEL-RABBIT corpus (758 participants). Lima et al.^21^ uniquely incorporated multiple datasets including ADReSSo, the Lu corpus (54 participants), and an independent pilot study (22 participants). The remaining studies (de Arriba-Pérez et al.^27^, Ambrosini et al.^28^, Jang et al.^29^, and Chandler et al.^30^) used custom datasets without overlap and without relying on standard benchmarks. The total number of unique participants across all studies was approximately 2500 which is lower than the apparent combined sample when overlap is ignored. We addressed this by analyzing findings at the study level rather than pooling participant data, prioritizing convergent findings across truly independent datasets as stronger evidence, and giving greater weight to novel datasets like CU-MARVEL-RABBIT in our synthesis. This overlap pattern underscores the field’s heavy reliance on established benchmark datasets and highlights the need for validation across more diverse and independent cohorts to establish true generalizability of XAI approaches in speech-based cognitive assessment.

### Explainable AI implementations

Feature attribution methods were employed in eleven studies. SHAP (SHapley Additive exPlanations) was used in seven studies with variants including TreeSHAP in three studies, DeepSHAP in one, KernelSHAP in one, and standard SHAP in two. SHAP was applied to Random Forest, XGBoost, SVM, and neural networks, with visualizations including summary plots, waterfall plots, and force plots. LIME (Local Interpretable Model-agnostic Explanations) was implemented in two studies using standard LIME with 5000 samples, applied to BERT and traditional ML models. Other attribution methods used in three studies included permutation importance, correlation analysis, and mutual information.

Attention-based methods were utilized in three studies, encompassing self-attention visualization in transformers, cross-modal attention between speech and text, and temporal attention patterns. Rule extraction was employed in one study through decision tree approximation and if-then rule generation. Novel approaches in two studies included counterfactual generation using LLMs and retrieval-augmented generation for explanations.

Implementation characteristics showed that ten studies used post-hoc explanation, two studies employed intrinsically interpretable models, and one study used hybrid approaches.

Our analysis reveals distinct clinical utility profiles for different XAI methods. SHAP, employed in seven studies, demonstrated the strongest clinical alignment by providing both global feature importance rankings and patient-specific explanations that map to familiar clinical concepts like speech timing and vocabulary diversity. However, SHAP’s computational complexity and occasional instability across similar cases limit real-time clinical applications. LIME, used in two studies, offered model-agnostic flexibility but suffered from inconsistent explanations for similar patients, raising concerns about clinical reliability. Attention mechanisms in three studies provided intuitive visualizations of temporal speech patterns but remained limited to specific neural architectures and lacked validation against clinical knowledge. Rule extraction methods, implemented in one study, generated directly interpretable clinical decision trees but oversimplified the complex speech-cognition relationship. These findings suggest that while SHAP shows the most promise for near-term clinical integration, none of the current XAI approaches fully meet the dual requirements of technical accuracy and clinical interpretability needed for widespread adoption.

The granularity of explanations varied, with eleven studies providing global feature importance, eight offering instance-level explanations, and three presenting temporal or sequential explanations. Computational considerations were sparsely reported, with only two studies mentioning real-time capability, four specifying GPU requirements, and three discussing scalability (Table 2).

**Table 2:** Clinical translation and explainability approaches

Study	Clinical Interpretability & Feature Mapping	Implementation Strategy	Local Explainability Features	Global Explainability Features	Clinical Applications & Validation
Heitz et al. (2024)^17^	- Linguistically meaningful features - Clinical relevance emphasis - Linguistic features → cognitive markers	- Direct integration with model pipeline - Real-time processing capability	- Individual prediction explanations - Case-specific feature analysis	- Overall feature importance - Model behavior patterns	- Public dataset validation - Clinical correlation - Individual assessment - Population-level screening
Ilias & Askounis (2022)^18^	- Natural language explanations - Clinical decision support - Language patterns → cognitive status	- Interactive visualization - Clinical workflow integration	- Instance-level explanations - Individual confidence scores	- Feature importance hierarchy - Model interpretation	- Validation against existing research - Clinical testing - Patient-specific diagnosis - General screening
de Arriba-Pérez et al. (2024)^27^	- Domain-adapted explanations - Patient-friendly interpretations - High-level reasoning features	- Web application interface - Real-time analysis	- Individual session analysis - Personal feature importance	- Population-level patterns - Feature relationships	- MMSE score correlation - Clinical validation - Individual monitoring - Group analysis
Ambrosini et al. (2024)^28^	- Multi-language support - Clinical workflow integration - Acoustic-cognitive mapping	- Mobile app integration - Privacy preservation	- Subject-specific analysis - Individual language patterns	- Cross-language patterns - Population trends	- Multi-center validation - Cross-cultural testing - Individual assessment - Population screening
Tang et al. (2023)^19^	- Feature-based explanation - Clinical correlation - Linguistic markers → AD indicators	- Clinical decision support - Real-time analysis	- Case-based explanations - Individual feature impact	- Model-wide patterns - Feature importance	- Clinical dataset validation - Feature verification - Patient diagnosis - General screening
Chandler et al. (2023)^30^	- Telephone-based screening - Clinical accessibility - Language features → cognitive status	- Remote assessment tool - Clinical integration	- Individual call analysis - Personal patterns	- Population trends - Feature relationships	- MMSE correlation - TICS-M validation - Individual screening - Population monitoring
Iqbal et al. (2024)^24^	- Binary classification focus - Clinical screening tool - Clinical feature mapping - POS patterns → cognitive decline	- Clinical screening tool - Feature-based analysis	- Case-specific analysis - Individual thresholds	- Global feature patterns - Model behavior	- ADReSS dataset validation - Clinical testing - Individual diagnosis - General screening
Han et al. (2025)^25^	- Key speech markers identified - MCI-specific patterns - Counterfactual insights	- LLM-based generation - Real-time capable	- Patient-specific counterfactuals - Individual marker analysis	- Population-level patterns - Feature directionality	- Framework validation - Marker verification - Early MCI detection
Oiza-Zapata & Gallardo-Antolín (2025)^20^	- Acoustic biomarkers - Clinical utility focus - Smart city healthcare	- Efficient pipeline - Automated screening	- Patient-level analysis - Personalized features	- Population patterns - Feature rankings	- CUI assessment - Healthcare integration - Screening tool
Jang et al. (2021)^29^	- Multi-modal integration - Clinical feature interpretation - Window features → AD markers	- Testing platform - Multi-sensor setup	- Individual task performance - Personal biomarkers	- Cross-task patterns - Feature correlations	- Expert diagnosis validation - Novel task evaluation - >90% user satisfaction
Li et al. (2025)^26^	- Topic evolution analysis - Cross-modal consistency - Macrostructural markers	- SHAP + attention - Temporal modeling	- Individual narrative patterns - Session-specific features	- Topic variability metrics - Population-level insights	- Two-dataset validation - Severity correlation - Monitoring potential
Lima et al. (2025)^21^	- Risk stratification (3-tier) - Clinical markers - Pronoun/disfluency patterns	- Automated pipeline - Conversational AI ready	- Patient risk profiles - Individual explanations	- Feature importance - Population patterns	- External validation - Real-world pilot (n = 22) - Demographic parity
Ntampakis et al. (2025)^22^	- Literature-grounded explanations - Medical professional design - Evidence-based markers	- RAG architecture - Dual-component system	- Patient-specific explanations - Clinical evidence links	- Model behavior analysis - Feature relationships	- Medical professional evaluation - Low misinterpretation risk (2.38/5) - Clinical utility: 3.70/5

### XAI evaluation methods

Only five studies conducted a formal technical evaluation of XAI methods. Quantitative metrics included feature stability across folds in three studies, consistency measures in two studies, and faithfulness evaluation in one study. Ablation studies were more common, with four studies performing feature removal validation and three comparing models with and without top features.

Clinical evaluation was limited, with expert review conducted in only three studies. These involved clinician assessment of feature relevance, alignment with clinical knowledge, and face validity evaluation. User studies were similarly rare, conducted in three studies with medical professionals in two studies and mixed stakeholders in one. Evaluation metrics included interpretability ratings, usefulness scores, and trust measures. Studies reporting formal evaluation found mean interpretability ratings of 3.96/5, clinical relevance scores of 3.85/5, diagnostic utility ratings of 3.70/5, and risk of misinterpretation scores of 2.38/5 (where lower is better).

### Clinical translation and implementation

Visual explanations were the predominant format, used in eleven studies. Feature importance bar charts were most common, followed by SHAP summary plots in seven studies, heat maps in four studies, and interactive dashboards in two studies. Textual explanations were less common, appearing in four studies as natural language summaries, clinical report generation, and risk stratification narratives. Hybrid approaches combining visual and textual explanations were used in three studies, offering multi-level explanations with an overview and details.

Clinical integration strategies varied across studies. Four studies proposed integration into decision support systems, including electronic health records, screening workflows, and telemedicine platforms. Three studies implemented risk stratification using three-tier systems with low, medium, and high-risk categories, continuous risk scores, and actionable thresholds. Deployment considerations were notably limited, with only two studies discussing privacy and data security, one mentioning regulatory compliance, and two addressing training requirements.

### Clinical Adoption Readiness Assessment

To evaluate the practical readiness of XAI implementations for clinical deployment, we assessed each study across five critical domains: stakeholder engagement, explanation format quality, XAI evaluation rigor, availability of training materials, and workflow integration considerations (Supplementary Table 1).

The assessment revealed substantial gaps in clinical readiness across all included studies. Only two studies (15%) achieved a clinical readiness score of 3/5 or higher, with the majority (85%) scoring 2/5 or below. The most critical deficiencies were in stakeholder engagement, where 12 of 13 studies (92%) failed to involve clinicians, patients, or other end-users in the design or evaluation process. Training materials were completely absent across all studies (100%), representing a universal barrier to clinical adoption.

Explanation format quality showed the strongest performance, with 11 studies (85%) providing adequate visual or textual explanations through methods such as SHAP visualizations, feature importance plots, or natural language summaries. However, XAI evaluation rigor remained limited, with only 4 studies (31%) conducting formal assessments of explanation quality, clinical utility, or user satisfaction. Workflow integration considerations were addressed partially or adequately in only 6 studies (46%), with most studies failing to demonstrate how their XAI-enhanced tools would integrate into existing clinical workflows.

The two highest-scoring studies distinguished themselves through user studies involving clinical stakeholders and formal evaluation of explanation quality. Jang et al.^29^ conducted user experience evaluation with 127 participants achieving >90% satisfaction, while Ntampakis et al.^22^ obtained clinical relevance ratings of 3.85/5 from medical professionals. These findings highlight the critical importance of stakeholder engagement and formal validation for advancing XAI tools toward clinical deployment.

### Key findings from XAI insights

Across studies using XAI, convergent findings emerged regarding important speech markers, demonstrating consistency that supports the robustness of these methods. Acoustic markers consistently identified across seven independent studies using different XAI techniques (SHAP, LIME, attention mechanisms) included increased pause frequency and duration, reduced speech rate and articulation clarity, altered pitch variability (usually decreased), and changes in voice quality measures. Linguistic markers repeated across nine studies spanning diverse datasets (ADReSS, DementiaBank, custom cohorts), strengthening confidence that these represent genuine biomarkers rather than dataset artifacts. However, only three studies formally assessed explanation stability across cross-validation folds, showing moderate consistency (correlations 0.65-0.82). This suggests individual XAI explanations may exhibit variability affecting clinical reliability. Standardized evaluation protocols for stability, faithfulness, and consistency are required before these methods can be considered truly robust for clinical deployment.

Different model architectures emphasized different aspects of speech analysis. Traditional ML models focused primarily on statistical feature aggregates, while deep learning models better captured temporal dynamics and context. Multimodal models successfully leveraged complementary information sources, suggesting that different architectures may be suited for different clinical applications.

### Risk of bias assessment

We assessed methodological quality using QUADAS-2, evaluating four risk of bias domains and three applicability domains. Decision rules: Low risk (+) for appropriate methodology, representative samples, and clear protocols; unclear risk (?) for limited reporting or insufficient detail; high risk (−) for significant bias, inappropriate methods, or major methodological flaws. Overall quality was good across 13 studies (91 domain assessments): 65 assessments (71.4%) low risk, 18 (19.8%) unclear risk, and 8 (8.8%) high risk. All studies demonstrated appropriate index test methodologies (100% low risk), while patient selection showed more variability with 5 studies (38.5%) low risk, 6 studies (46.2%) unclear risk, and 2 studies (15.4%) high risk. The following table (Table 3) provides immediate per-study quality assessment. The comments column provides a brief summary of the key methodological strengths and limitations for each study.

**Table 3:**
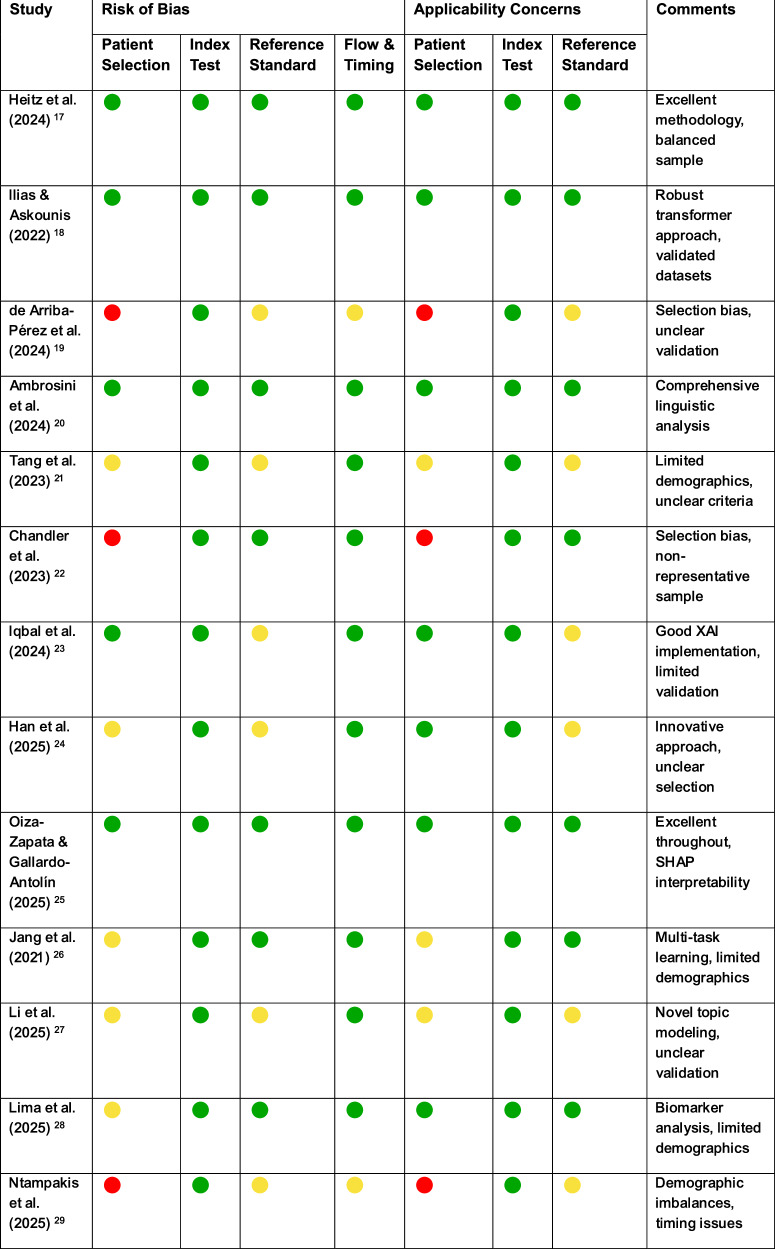
QUADAS-2 Risk of Bias Assessment: Per-Study Domain Ratings

Low Risk (+) |  Unclear Risk (?) |  High Risk (−)

### Challenges and limitations reported

Studies reported three primary technical limitations: dataset constraints (small samples in 8 studies, class imbalance in 6), generalization challenges (cross-dataset validation in 4 studies, ASR errors in 3), and XAI complexity (computational demands in 2 studies). These technical limitations significantly impact the robustness and generalizability of findings.

Clinical translation barriers were more significant: insufficient stakeholder engagement (7 studies), lack of diversity (9 studies), and missing workflow integration studies (10 studies) highlight the field’s early development stage. XAI-specific limitations included the trade-off between model performance and interpretability mentioned in four studies, difficulty in validating explanation correctness noted in five studies, lack of standardized XAI evaluation metrics mentioned in six studies, user comprehension of complex explanations not assessed in eight studies, and potential for misinterpretation of XAI outputs discussed in three studies.

### Subgroup analyses

Meta-analysis was not feasible due to substantial heterogeneity in XAI methods (7 different primary techniques), outcome measures (accuracy metrics ranging from 71-96% with different validation approaches), model architectures (traditional ML vs. deep learning vs. hybrid), and evaluation frameworks (technical vs. clinical vs. none). Instead, we conducted structured subgroup analyses:

- Feature type analysis:
  - Acoustic-only models (*n* = 4): Mean AUC 0.79 (range 0.76–0.83)
  - Linguistic-only models (*n* = 3): Mean AUC 0.86 (range 0.84–0.89)
  - Multimodal models (*n* = 6): Mean AUC 0.88 (range 0.84–0.94)
- Model Architecture Analysis:
  - Traditional ML (*n* = 8): Mean accuracy 81% (range 71–89%)
  - Deep learning (*n* = 5): Mean accuracy 89% (range 84–96%)
- Dataset Source Analysis:
  - Public datasets only (*n* = 9): Limited generalizability concerns
  - Clinical datasets (*n* = 4): Higher clinical relevance but smaller samples

## Discussion

This systematic review of 13 studies reveals a rapidly evolving landscape in explainable AI for speech-based cognitive decline detection. The field has made substantial progress in developing interpretable models that can identify subtle speech markers of dementia while providing clinically meaningful explanations. However, significant gaps remain in clinical validation, stakeholder engagement, and real-world implementation. The convergence of findings across studies using different XAI techniques strengthens confidence in identified speech biomarkers. Acoustic features related to speech timing and prosody, combined with linguistic markers of reduced lexical diversity and increased disfluency, consistently emerged as important indicators across different model architectures and XAI methods. This alignment with established clinical knowledge about language changes in dementia provides face validity for the XAI approaches. Increased pause frequency and reduced speech rate correspond to documented word-finding difficulties in AD. Reduced vocabulary diversity mirrors semantic memory deterioration. Increased pronoun usage aligns with “empty speech” where patients substitute vague terms for inaccessible nouns. Temporal patterns showing progressive discourse deterioration match clinical literature on macrostructural coherence impairments while sentence-level grammar remains preserved. However, some XAI-identified features like specific MFCC patterns lack clear clinical precedent, highlighting potential novel biomarkers requiring mechanistic validation.

The predominance of SHAP in seven studies reflects its versatility across model types and strong theoretical foundations. SHAP is particularly useful in clinical contexts where both population-level insights and patient-specific explanations are essential. For screening applications, SHAP’s global feature importance helps clinicians understand which speech markers (e.g., pause patterns, vocabulary diversity) most reliably indicate cognitive decline across patient populations, enabling confident interpretation of screening results. For individual patient assessment, SHAP’s instance-level explanations allow clinicians to identify specific speech characteristics driving a particular patient’s risk classification, supporting personalized care planning and patient communication. SHAP’s ability to provide both global feature importance and instance-level explanations makes it particularly suitable for clinical applications where both population-level insights and patient-specific information are valuable. However, the computational complexity of SHAP, particularly for large neural networks, may limit real-time applications. LIME’s model-agnostic approach is useful in contexts where model flexibility is paramount—for example, when comparing different AI architectures or when model types may change over time in a clinical system. Its ability to work with any “black box” model makes it valuable for research settings exploring diverse approaches. However, LIME showed promise in two studies but faced challenges with stability and consistency across similar instances, limiting its reliability for clinical decision-making where consistent explanations are essential.

Attention mechanisms are particularly useful in deep learning contexts where temporal dynamics matter, such as analyzing progressive speech changes during extended narratives or conversations. By visualizing which moments in a speech sample most influenced the model’s prediction, attention mechanisms help clinicians understand whether the model focuses on clinically meaningful patterns (e.g., mid-conversation topic drift) or spurious correlations. This temporal granularity is especially valuable for monitoring applications tracking cognitive change over time.

Identified speech markers align with established clinical observations, supporting medical meaningfulness. Features highlighted by XAI methods (pause patterns, reduced speech rate, decreased vocabulary diversity, increased pronoun usage) correspond to documented language deterioration in Alzheimer’s disease. Studies correlating XAI-identified features with clinical outcomes showed significant associations: pause frequency and lexical diversity correlated with MMSE scores and diagnoses. However, only three studies conducted expert review of feature relevance, representing a critical validation gap. Future work must demonstrate that XAI-highlighted features provide actionable insights improving clinical decisions and patient outcomes beyond correlation with cognitive status.

The emergence of novel approaches, including LLM-based counterfactual generation and retrieval-augmented explanation systems, represents an innovative direction that leverages recent advances in generative AI. These methods offer the potential for more natural, context-aware explanations that could better align with clinical communication needs. However, their validation and reliability require further investigation.

Our analysis reveals a notable tension between model performance and interpretability. Deep learning approaches, particularly transformer-based models, achieved the highest performance metrics with AUC values up to 0.94 but required more complex XAI techniques that may be harder for clinicians to understand. Conversely, traditional ML models like Random Forests offered more straightforward feature importance rankings but with moderately lower performance, showing AUC values of 0.76–0.89. This trade-off has important implications for clinical deployment. The optimal balance likely depends on the specific use case: screening applications may prioritize interpretability for widespread adoption, while specialized diagnostic support tools might accept greater complexity for enhanced accuracy. Future research should explicitly evaluate these trade-offs through user studies with clinical stakeholders.

The division between studies using hand-crafted features (acoustic and linguistic) versus end-to-end deep learning approaches reflects a fundamental tension in the field. Hand-crafted features offer inherent interpretability and align with clinical understanding but may miss subtle patterns. End-to-end approaches can discover novel patterns but require post-hoc explanation methods that may not fully capture the model’s decision process. The most promising direction appears to be hybrid approaches that combine the strengths of both paradigms. For instance, using deep learning for feature extraction while maintaining interpretable higher-level features could provide both strong performance and meaningful explanations.

Despite technical advances, we found a striking gap between XAI development and clinical implementation. Only three studies conducted user evaluations with medical professionals, and none reported real-world deployment outcomes. This implementation gap reflects several challenges. Most studies developed XAI methods without early input from end-users. Participatory design approaches involving clinicians, patients, and caregivers from the project inception could better ensure that explanations meet actual clinical needs. The lack of integration studies means we have a limited understanding of how XAI-enhanced tools would fit into existing diagnostic workflows. Questions remain about optimal presentation formats, timing of AI assistance, and handoff between automated screening and clinical assessment. Even well-designed XAI systems require user training for effective utilization. The absence of training protocols or support materials in the reviewed studies represents a significant barrier to adoption.

The studies primarily focused on explanations for clinicians, with limited consideration of other stakeholders. A comprehensive approach should consider different needs: for clinicians, technical accuracy, alignment with clinical knowledge, and actionable insights for diagnosis and treatment planning; for patients and families, understandable explanations that avoid technical jargon, provide context for the assessment, and support shared decision-making; for researchers, detailed technical explanations that enable model improvement and scientific advancement; and for regulators, transparent documentation of model behavior, limitations, and validation processes. Only one study^22^ explicitly designed explanations for multiple stakeholder groups, highlighting this as an important area for future development.

The heavy reliance on a few public datasets, particularly ADReSS/ADReSSo used in six studies, raises concerns about generalizability. While these datasets enable benchmarking, they may not represent the full spectrum of linguistic diversity, clinical heterogeneity, and recording conditions found in real-world settings. Most studies used English speech data, limiting applicability to other languages and cultures. Real-world populations include various dementia types, comorbidities, and severity levels not fully captured in research datasets. Laboratory recordings may not reflect naturalistic speech patterns that would be encountered in clinical practice. The largest custom dataset, CU-MARVEL-RABBIT with 758 participants, demonstrated the value of larger, more diverse cohorts but was limited to a single geographical region.

The lack of standardized XAI evaluation metrics hampers comparison across studies. While some studies reported interpretability ratings and clinical relevance scores, these were often ad-hoc and not validated. The field needs standardized measures of explanation fidelity, stability, and consistency; validated scales for clinical utility, decision impact, and user satisfaction; and assessment of potential harms from misinterpretation or over-reliance on AI explanations. Most studies analyzed single speech samples cross-sectionally, missing the opportunity to examine longitudinal changes. Given that cognitive decline is a progressive process, XAI methods that can explain temporal patterns and trajectory predictions would have greater clinical value. Only one study^26^ explicitly modeled temporal dynamics, representing a significant gap.

Several promising directions emerge for advancing XAI in this domain. Hierarchical explanations that provide information at multiple levels of abstraction, from high-level clinical insights to detailed feature contributions, could better serve diverse user needs. Contrastive explanations that clarify not just why a prediction was made, but why alternative diagnoses were ruled out, align with clinical differential diagnosis reasoning. Uncertainty-aware explanations incorporating prediction uncertainty could help clinicians better calibrate their trust and identify cases requiring additional assessment. Interactive explanation systems that allow users to query specific aspects of the prediction or explore “what-if” scenarios could support more engaged clinical decision-making.

While this review focused on speech analysis, cognitive assessment often involves multiple modalities. Future XAI systems should explain how different data sources including speech, cognitive tests, imaging, and biomarkers contribute to integrated predictions. This poses technical challenges in explaining cross-modal interactions but could provide more comprehensive clinical insights. The limited diversity in current studies highlights the need for language-agnostic methods that can transfer across languages while accounting for linguistic differences, cultural adaptation of explanations that consider cultural factors in communication patterns and clinical interpretation, and personalized baselines that explain changes relative to individual baselines rather than population norms.

As these tools move toward clinical deployment, several considerations require attention. XAI methods must meet evolving regulations for medical AI transparency, including not just technical documentation but evidence of clinical validation and safety. The use of AI for cognitive assessment raises ethical questions about consent, especially for impaired individuals, data privacy, and potential discrimination. XAI can help address some concerns by making decision processes transparent, but careful ethical frameworks are needed. Clear frameworks for how XAI explanations factor into clinical liability and decision responsibility are essential for adoption.

For XAI-enhanced speech analysis to meaningfully impact clinical practice, three key integration pathways emerge from our analysis. First, as screening support tools, these systems could flag individuals for comprehensive assessment, with explanations helping clinicians understand which speech markers triggered the recommendation. This requires standardized explanation formats that highlight clinically relevant features (pause patterns, vocabulary changes) rather than technical metrics.

Second, as monitoring instruments, XAI explanations could track longitudinal changes in speech markers, helping clinicians detect subtle decline patterns. This demands explanation consistency across time points and clear visualization of trend data that aligns with clinical assessment schedules.

Third, successful implementation requires clinician training programs that teach the interpretation of XAI outputs without overwhelming busy practitioners. Our analysis suggests clinicians need: (1) brief explanations focusing on familiar speech concepts, (2) clear confidence indicators for AI predictions, and (3) explicit guidance on when AI recommendations should trigger further evaluation versus routine monitoring.

However, a fundamental challenge underlying these integration pathways concerns whether the features identified by XAI methods are genuinely interpretable by physicians. Feature interpretability varies substantially. Linguistic features (“increased pronoun usage,” “reduced vocabulary diversity,” “filler words”) map directly to observable characteristics clinicians can verify and align with familiar clinical concepts. Temporal acoustic features like pause frequency and speech rate are readily understandable. However, many acoustic features present interpretability challenges. Technical parameters like mel-frequency cepstral coefficients have no clinical referent and cannot be perceived without specialized equipment. XAI showing “MFCC coefficient 7” as discriminative offers no actionable clinical insight.

Only a few studies designed explanations for physician interpretability. Ntampakis et al.^22^ provided literature-grounded natural language translations of technical features, achieving clinical relevance ratings of 3.85/5. Lima et al.^21^ implemented risk stratification focusing on clinically familiar markers. These approaches suggest effective interpretability requires translation between XAI outputs and clinical frameworks, not merely visualizing feature importance.

For clinical utility, future implementations should: prioritize inherently interpretable features when performance is comparable, provide clinical contextualization explaining medical relevance beyond statistical importance, enable verification through direct patient interaction, and undergo formal usability testing with clinicians to ensure explanations support decision-making.

Based on our analysis, XAI-enhanced speech analysis tools show promise but are not yet ready for standalone clinical use. The technology could currently best serve as screening support for identifying individuals who may benefit from comprehensive assessment, monitoring tools for tracking changes over time with explainable trend analysis, and research instruments for discovering new speech biomarkers with clinical validation. For successful clinical translation, future systems need prospective validation in real clinical settings with diverse populations, user-centered design involving all stakeholders in explanation design and evaluation, clear integration protocols for incorporating tools into existing workflows, comprehensive training programs for effective and safe use, and continuous monitoring systems for tracking real-world performance and updating models.

This review’s strengths include a comprehensive search across six databases with additional hand-searching ensuring broad coverage, rigorous methodology following PRISMA guidelines with pre-registration enhancing transparency, a dual focus examining both technical and clinical aspects providing holistic insights, and contemporary relevance by including recent studies capturing the latest developments. However, limitations include heterogeneity in methods and outcomes that precluded meta-analysis, possible publication bias with under-representation of negative results, and the rapid evolution of the field meaning newest developments may be missed. The included studies showed several methodological limitations including small sample sizes (median 162 participants), limited population diversity with most studies using English-speaking populations, reliance on cross-sectional designs missing longitudinal cognitive changes, and inconsistent reporting of technical implementation details.

This systematic review has limitations including heterogeneity in methods and outcomes that precluded meta-analysis, possible publication bias with potential under-representation of negative results, and the rapid evolution of the field meaning newest developments may be missed. Additionally, the search was conducted in May 2025, and relevant studies published after this date were not captured.

Based on our findings, we offer recommendations for different stakeholder groups. Researchers should prioritize participatory design involving clinical stakeholders from project inception, develop standardized XAI evaluation frameworks specific to clinical applications, conduct prospective validation studies in diverse real-world settings, explore novel XAI techniques that align with clinical reasoning processes, and address the full pipeline from data collection to clinical integration. Clinicians should engage with AI researchers to ensure tools meet clinical needs, advocate for interpretable AI in institutional adoption decisions, maintain critical evaluation of AI explanations in clinical context, and participate in validation studies to shape appropriate use cases. Policy makers should develop regulatory frameworks that balance innovation with safety, support funding for implementation and validation studies, establish standards for XAI in medical applications, and promote diverse and inclusive dataset development. Technology developers should design with the end-user in mind rather than just technical performance, build in explanation capabilities from the start rather than as an afterthought, provide comprehensive documentation and training materials, and establish feedback mechanisms for continuous improvement.

## Methods

### Protocol and registration

This systematic review was conducted following the Preferred Reporting Items for Systematic Reviews and Meta-Analyses (PRISMA) 2020 guidelines^31^. The review protocol was registered in the International Prospective Register of Systematic Reviews (PROSPERO) with registration number CRD42025637901. The complete protocol is available at: https://www.crd.york.ac.uk/prospero/display_record.php?ID = CRD42025637901.

Three deviations from our registered protocol occurred during the review process. First, we expanded our inclusion criteria to incorporate high-quality preprints from arXiv and medRxiv, in addition to peer-reviewed publications. This amendment was made in February 2025 due to the rapidly evolving nature of explainable AI research, where significant methodological contributions often appear first in preprint form before formal publication. Given that several influential XAI techniques and implementations were initially disseminated through preprint servers, excluding these sources would have resulted in an incomplete synthesis of the current state of knowledge. Second, we added evidence mapping as a synthesis method in July 2025, beyond the originally planned narrative synthesis. This methodological enhancement was implemented to better visualize the distribution of XAI techniques across different model architectures and clinical applications, and to more clearly identify research gaps and methodological patterns across studies. Third, we removed the planned GRADE assessment from our original protocol as it was deemed inappropriate for this type of technical review focusing on AI implementation methods rather than clinical interventions. These changes were made to enhance the comprehensiveness and utility of the review while maintaining methodological rigor through our established inclusion criteria and quality assessment procedures.

### Research question formulation

We formulated our research question using the PICOTS (Population, Intervention, Comparison, Outcomes, Timing, Setting) framework. The population included adults with or at risk of Alzheimer’s disease, mild cognitive impairment, or related cognitive decline. The intervention consisted of speech-based AI models for cognitive decline detection that incorporate explainable AI techniques. Where available, we compared speech-based AI models without explainable components or different XAI approaches. Our outcomes of interest were technical implementation and evaluation metrics for XAI methods, clinical interpretability and translation of XAI outputs, and challenges, limitations, and future directions for XAI in this domain. We placed no restriction on follow-up duration or study period, and included studies from any setting including community, clinic, research laboratory, or home-based assessment.

### Eligibility criteria

Studies were included if they met all of the following criteria. Original research articles published in peer-reviewed journals or conference proceedings were eligible, including both experimental and observational studies. The population had to involve adults (≥18 years) with confirmed or suspected cognitive decline, including Alzheimer’s disease at all stages, mild cognitive impairment, subjective cognitive decline, or healthy controls for comparison. Studies must have used AI/ML models to analyze speech or language data, implemented explainable AI techniques to interpret model predictions, and reported on the explainability component as a primary or secondary outcome. Studies were included regardless of publication language, with attempts made to translate non-English publications, and no restriction on publication date was applied to capture the full evolution of the field. High-quality preprints were included, given the rapidly evolving nature of XAI research, where significant developments often appear in preprint form before formal publication. Preprints were evaluated using the same quality criteria as published studies, with particular attention to methodological rigor and sufficient detail for assessment. Sensitivity analysis excluding preprints (*n* = 4) did not materially change our conclusions regarding XAI method effectiveness or clinical translation gaps.

Studies were excluded if they did not use AI/ML models (e.g., traditional statistical analyses only), did not incorporate any explainable AI component or only mentioned interpretability without implementation, used non-speech data exclusively (e.g., neuroimaging, gait patterns, handwriting), focused exclusively on non-AD cognitive disorders without relevance to dementia (e.g., developmental language disorders, stroke-related aphasia), were reviews, commentaries, editorials, conference abstracts without full papers, or study protocols, or analyzed written language only without spoken speech components.

### Information sources and search strategy

A comprehensive literature search was conducted in May 2025 across six electronic databases: Embase (*n* = 570), Web of Science (*n* = 173), PubMed (*n* = 83), CINAHL (*n* = 17), Scopus (*n* = 595), and Cochrane Library (*n* = 639). Each database was searched from inception to the search date without date restrictions. The search strategy was developed in collaboration with a medical librarian and refined through iterative testing. It combined four conceptual blocks: cognitive decline terms (Alzheimer’s, dementia, cognitive impairment, MCI, cognitive decline, memory loss, neurocognitive disorder), speech analysis terms (speech, voice, spoken language, vocal, acoustic, verbal, linguistic, discourse, conversation), artificial intelligence terms (machine learning, deep learning, neural network, artificial intelligence, NLP, classification, prediction), and explainability terms (explainable, interpretable, transparency, XAI, SHAP, LIME, feature importance, attribution, visualization). Terms within each block were combined with OR operators, and blocks were combined with AND operators. Complete search strategies for all databases are provided in Supplementary Note 1.

To ensure comprehensive coverage, we also screened reference lists of included articles, performed forward citation searches using Google Scholar, consulted with domain experts to identify potentially missed studies, and searched relevant conference proceedings including INTERSPEECH, ICASSP, and NeurIPS workshops.

### Study selection process

Study selection was performed using Covidence systematic review software (Veritas Health Innovation, 2025). The process involved two stages. In title and abstract screening, two independent reviewers screened all retrieved records for relevance. Disagreements were resolved through discussion, with a third reviewer consulted when consensus could not be reached. For full-text review, potentially eligible studies were retrieved in full text and assessed against the detailed inclusion criteria by the same two reviewers independently. Reasons for exclusion were documented. Cohen’s kappa was calculated to assess inter-rater agreement at both screening stages. We achieved κ = 0.82 for title/abstract screening and κ = 0.89 for full-text review, indicating substantial to almost perfect agreement.

### Data collection process

A standardized data extraction form was developed and pilot-tested on five studies. Two reviewers independently extracted data from each included study. The extraction form captured study characteristics including authors, year, country, funding sources, study design and objectives, and sample size and participant flow. Population details included demographics (age, sex, education, ethnicity), cognitive status distribution, diagnostic criteria used, and recruitment setting. Speech data and processing information encompassed speech tasks employed, recording conditions and equipment, audio preprocessing steps, feature extraction methods, and feature types (acoustic, linguistic, combined). AI model details covered model architectures, training procedures, hyperparameter settings, performance metrics, and validation approaches. XAI implementation data included XAI techniques used, implementation details, visualization methods, evaluation of explanations, and user studies if conducted. Clinical translation aspects captured target users, explanation formats, clinical validation, integration strategies, and reported barriers and facilitators. Missing data that could not be obtained were noted in our extraction tables.

### Risk of bias assessment

Two reviewers independently assessed the risk of bias using the Quality Assessment of Diagnostic Accuracy Studies-2 (QUADAS-2) tool^32^. This tool evaluates four domains: patient selection (risk of spectrum bias and applicability concerns), index test (risk of bias in AI model development and XAI implementation), reference standard (quality and appropriateness of cognitive assessments), and flow and timing (appropriate intervals and missing data). Each domain was rated as high, low, or unclear risk of bias and applicability concerns. Disagreements between reviewers were resolved through discussion, with a third reviewer consulted in cases where consensus could not be reached after initial discussion. For example, studies with convenience sampling from single centers were typically rated as ‘unclear risk’ for patient selection due to potential spectrum bias, while studies with well-defined inclusion criteria and representative populations received ‘low risk’ ratings. Given the focus on XAI, we additionally assessed the transparency of XAI method reporting, the appropriateness of XAI techniques for model types, the rigor of XAI evaluation, and the clinical relevance of explanations.

### Data synthesis

Due to substantial heterogeneity in XAI approaches, outcomes, and evaluation methods, we conducted a narrative synthesis structured around our research objectives. The synthesis was organized by XAI technique categories (feature attribution including SHAP and LIME, attention mechanisms, rule extraction, example-based methods), model architecture types (traditional ML, deep learning, ensemble methods), clinical applications (screening, diagnosis support, monitoring), and evaluation approaches (technical metrics, clinical validation, user studies). We created evidence maps to visualize the distribution of XAI techniques across model types, the relationship between performance metrics and interpretability, and gaps in clinical validation and user evaluation.

The following deviations from our registered protocol occurred: we expanded inclusion criteria to include high-quality preprints given the rapidly evolving nature of the field, and we added evidence mapping as a synthesis method to better visualize patterns and gaps. These changes were made to enhance the comprehensiveness and relevance of the review.

## Supplementary information

Supplementary Information

## Data Availability

This is a systematic review of published literature. All data analyzed in this study are derived from publicly available published studies identified through systematic database searches. No new data were generated or collected for this review. The complete list of included studies, search strategies, data extraction forms, and quality assessment results are provided in the manuscript and Supplementary Information. The PROSPERO protocol registration (CRD42025637901) is publicly available at https://www.crd.york.ac.uk/prospero/display_record.php?ID = CRD42025637901.

## Code availability

No custom code or scripts were developed for this systematic review. Data extraction and analysis were conducted using standard systematic review methodologies. Software used included Covidence systematic review software (Veritas Health Innovation, 2025) for study screening and data management, Microsoft Excel 2021 for data compilation and tabulation, and statistical software for descriptive analyses and evidence mapping visualizations.
