# Supplementary material for: A systematic review of explainable artificial intelligence methods for speech-based cognitive decline detection
Source: NPJ Digit Med. 2025 Nov 26;8:724. doi: 10.1038/s41746-025-02105-z (PMC12657886; doi:10.1038/s41746-025-02105-z)
Supplement: Supplementary file 1 — Supplementary Information [file 41746_2025_2105_MOESM1_ESM.pdf]

Supplementary Note 1: Complete Database Search Strategies

PubMed (MEDLINE) - Last searched: May 31, 2025 (n=83)

("explainable AI" OR "explainable artificial intelligence" OR "XAI" OR "explainability" OR "interpretable AI" OR "interpretable machine learning" OR "interpretability" OR "transparent AI" OR "transparent ML" OR "model transparency" OR "post-hoc explainability" OR "explanation by example" OR "feature importance" OR "feature attribution" OR "sensitivity analysis" OR "layer-wise relevance propagation" OR "DeepLIFT" OR "SHAP" OR "LIME" OR "counterfactual explanations" OR "saliency map" OR "saliency maps" OR "grad-CAM" OR "integrated gradients")

AND

("speech" OR "voice" OR "spoken language" OR "vocal" OR "audio" OR "speaking" OR "acoustic" OR "verbal" OR "verbal fluency" OR "verbal communication" OR "linguistic" OR "spontaneous speech" OR "discourse" OR "dialogue" OR "monologue" OR "narration" OR "reading aloud" OR "picture description" OR "phonetic" OR "phonological" OR "prosodic" OR "paralinguistic" OR "spoken conversation" OR "speech-based" OR "voice-based")

AND

("cognitive decline" OR "cognitive impairment" OR "cognitive dysfunction" OR "dementia" OR "Alzheimer's disease" OR "Alzheimer disease" OR "AD" OR "mild cognitive impairment" OR "MCI" OR "cognitive aging" OR "cognitive deficit" OR "cognitive screening" OR "cognitive assessment" OR "memory loss" OR "memory impairment" OR "memory decline" OR "memory complaint" OR "subjective cognitive decline" OR "preclinical dementia" OR "prodromal dementia" OR "Parkinson's disease" OR "Parkinson's disease dementia" OR "Lewy body dementia" OR "vascular dementia" OR "frontotemporal dementia")

Filters: None applied

Language restrictions: None

Publication date: No restrictions (inception to May 31, 2025)

Embase - Last searched: May 31, 2025 (n=570)

('explainable artificial intelligence'/exp OR 'artificial intelligence'/exp OR 'machine learning'/exp OR 'deep learning'/exp OR (explainable NEXT/1 (AI OR 'artificial intelligence')):ab,ti OR XAI:ab,ti OR (interpretable NEXT/1 (AI OR 'artificial intelligence' OR 'machine learning')):ab,ti OR interpretability:ab,ti OR (transparent NEXT/1 (AI OR

ML OR 'machine learning')):ab,ti OR 'model transparency':ab,ti OR 'post-hoc explainability':ab,ti OR 'feature importance':ab,ti OR 'feature attribution':ab,ti OR SHAP:ab,ti OR LIME:ab,ti OR 'counterfactual explanation*':ab,ti OR 'saliency map*':ab,ti OR 'integrated gradients':ab,ti OR 'layer-wise relevance':ab,ti OR DeepLIFT:ab,ti OR 'grad-CAM':ab,ti)

AND

('speech'/exp OR 'voice'/exp OR 'speech analysis'/exp OR 'speech recognition'/exp OR 'acoustic analysis'/exp OR 'phonetics'/exp OR 'linguistics'/exp OR 'verbal behavior'/exp OR speech:ab,ti OR voice:ab,ti OR (spoken NEXT/1 language):ab,ti OR vocal:ab,ti OR audio:ab,ti OR speaking:ab,ti OR acoustic:ab,ti OR verbal:ab,ti OR (verbal NEXT/1 (fluency OR communication)):ab,ti OR linguistic:ab,ti OR (spontaneous NEXT/1 speech):ab,ti OR discourse:ab,ti OR dialogue:ab,ti OR monologue:ab,ti OR narration:ab,ti OR (reading NEXT/1 aloud):ab,ti OR (picture NEXT/1 description):ab,ti OR phonetic:ab,ti OR phonological:ab,ti OR prosodic:ab,ti OR paralinguistic:ab,ti OR (speech NEXT/1 based):ab,ti OR (voice NEXT/1 based):ab,ti)

AND

('dementia'/exp OR 'Alzheimer disease'/exp OR 'mild cognitive impairment'/exp OR 'cognitive defect'/exp OR 'memory disorder'/exp OR 'neurocognitive disorder'/exp OR dementia:ab,ti OR Alzheimer*:ab,ti OR (cognitive NEXT/1 (decline OR impairment OR dysfunction OR deficit OR aging)):ab,ti OR MCI:ab,ti OR (mild NEXT/1 cognitive NEXT/1 impairment):ab,ti OR (memory NEXT/1 (loss OR impairment OR decline OR complaint)):ab,ti OR (subjective NEXT/1 cognitive NEXT/1 decline):ab,ti OR (preclinical OR prodromal NEXT/1 dementia):ab,ti OR (Parkinson* NEXT/1 disease):ab,ti OR (Lewy NEXT/1 body NEXT/1 dementia):ab,ti OR (vascular NEXT/1 dementia):ab,ti OR (frontotemporal NEXT/1 dementia):ab,ti)

Filters: None applied

Language: All languages

Date range: No restrictions

Web of Science (Core Collection) - Last searched: May 31, 2025 (n=173)

TS=(("explainable AI" OR "explainable artificial intelligence" OR "XAI" OR "explainability" OR "interpretable AI" OR "interpretable machine learning" OR "interpretability" OR "transparent AI" OR "transparent ML" OR "model transparency" OR "post-hoc explainability" OR "feature importance" OR "feature attribution" OR "SHAP" OR "LIME" OR "counterfactual explanation*" OR "saliency map*" OR "integrated

gradients" OR "layer-wise relevance" OR "DeepLIFT" OR "grad-CAM") AND ("speech" OR "voice" OR "spoken language" OR "vocal" OR "audio" OR "acoustic" OR "verbal" OR "verbal fluency" OR "verbal communication" OR "linguistic" OR "spontaneous speech" OR "discourse" OR "dialogue" OR "monologue" OR "narration" OR "reading aloud" OR "picture description" OR "phonetic" OR "phonological" OR "prosodic" OR "paralinguistic" OR "speech-based" OR "voice-based") AND ("cognitive decline" OR "cognitive impairment" OR "cognitive dysfunction" OR "dementia" OR "Alzheimer* disease" OR "AD" OR "mild cognitive impairment" OR "MCI" OR "cognitive aging" OR "memory loss" OR "memory impairment" OR "subjective cognitive decline" OR "preclinical dementia" OR "prodromal dementia" OR "Parkinson* disease" OR "Lewy body dementia" OR "vascular dementia" OR "frontotemporal dementia"))

Indexes: SCI-EXPANDED, SSCI, A&HCI, CPCI-S, CPCI-SSH, ESCI

Timespan: All years (1900-2025)

Language: All languages

CINAHL Complete - Last searched: May 31, 2025 (n=17)

(MH "Artificial Intelligence+" OR MH "Machine Learning+" OR TI ("explainable AI" OR "XAI" OR "interpretable AI" OR "SHAP" OR "LIME" OR "feature importance") OR AB ("explainable AI" OR "XAI" OR "interpretable AI" OR "SHAP" OR "LIME" OR "feature importance"))

AND

(MH "Speech+" OR MH "Voice+" OR MH "Speech Disorders+" OR MH "Language+" OR TI (speech OR voice OR "spoken language" OR vocal OR acoustic OR verbal OR linguistic OR discourse) OR AB (speech OR voice OR "spoken language" OR vocal OR acoustic OR verbal OR linguistic OR discourse))

AND

(MH "Dementia+" OR MH "Alzheimer's Disease+" OR MH "Cognition Disorders+" OR TI (dementia OR alzheimer* OR "cognitive decline" OR "cognitive impairment" OR MCI OR "mild cognitive impairment") OR AB (dementia OR alzheimer* OR "cognitive decline" OR "cognitive impairment" OR MCI OR "mild cognitive impairment"))

Limiters: None applied

Expanders: Apply equivalent subjects, Apply related words

Interface: EBSCOhost

Scopus - Last searched: May 31, 2025 (n=595)

TITLE-ABS-KEY(("explainable AI" OR "explainable artificial intelligence" OR "XAI" OR "explainability" OR "interpretable AI" OR "interpretable machine learning" OR "interpretability" OR "transparent AI" OR "model transparency" OR "feature importance" OR "feature attribution" OR "SHAP" OR "LIME" OR "counterfactual explanation*" OR "saliency map*") AND ("speech" OR "voice" OR "spoken language" OR "vocal" OR "audio" OR "acoustic" OR "verbal" OR "linguistic" OR "spontaneous speech" OR "discourse" OR "speech-based") AND ("cognitive decline" OR "cognitive impairment" OR "dementia" OR "Alzheimer*" OR "mild cognitive impairment" OR "MCI" OR "memory loss" OR "neurocognitive"))

Document type: All

Language: All

Subject area: All

Cochrane Library (Cochrane Reviews, CENTRAL, HTA, EED) - Last searched: May 31, 2025 (n=639)

#1 MeSH descriptor: [Artificial Intelligence] explode all trees

#2 ("explainable AI" OR "XAI" OR "interpretable AI" OR "SHAP" OR "LIME" OR "feature importance"):ti,ab,kw

#3 #1 OR #2

#4 MeSH descriptor: [Speech] explode all trees

#5 MeSH descriptor: [Voice] explode all trees

#6 (speech OR voice OR "spoken language" OR vocal OR acoustic OR verbal OR linguistic):ti,ab,kw

#7 #4 OR #5 OR #6

#8 MeSH descriptor: [Dementia] explode all trees

#9 MeSH descriptor: [Alzheimer Disease] explode all trees

#10 MeSH descriptor: [Cognitive Dysfunction] explode all trees

#11 (dementia OR alzheimer* OR "cognitive decline" OR "cognitive impairment" OR MCI):ti,ab,kw

#12 #8 OR #9 OR #10 OR #11

#13 #3 AND #7 AND #12

Publication Year: No restrictions

Additional Sources:

- Forward citation searching via Google Scholar for included articles
- Reference list screening of included studies and relevant systematic reviews
- Expert consultation with domain specialists in speech pathology and AI
- Conference proceedings: INTERSPEECH (2020-2025), ICASSP (2020-2025), NeurIPS workshops on healthcare AI (2020-2025)
- Preprint servers: arXiv (cs.CL, cs.AI, cs.LG sections), medRxiv, bioRxiv (searched May 31, 2025)

Supplementary Table 1: XAI Clinical Adoption Readiness Assessment

Study	Stakeholder Engagement	Explanation Format	XAI Evaluation	Training Materials	Workflow Integration	Clinical Readiness Score (/5)
Han et al. (2025)	○ (No clinician input)	◐ (Feature importance + counterfactuals)	◐ (Technical validation only)	○ (None mentioned)	○ (Not addressed)	1/5
Oiza-Zapata & Gallardo-Antolín (2025)	○ (No stakeholder input)	● (Visual + SHAP plots)	◐ (CUI assessment)	○ (None mentioned)	◐ (Smart city concept)	2/5
Jang et al. (2021)	● (User study n=127, >90% satisfaction)	◐ (Feature importance tables)	● (User experience evaluation)	○ (None mentioned)	● (Testing platform)	3/5
Li et al. (2025)	○ (No clinician input)	● (SHAP + attention + correlation plots)	◐ (Technical correlation analysis)	○ (None mentioned)	○ (Not addressed)	2/5
Lima et al. (2025)	○ (No stakeholder input)	● (Risk stratification + SHAP)	◐ (Demographic parity analysis)	○ (None mentioned)	◐ (Conversational AI ready)	2/5
Ntampakis et al. (2025)	● (Medical professional	● (RAG-based natural	● (Clinical validation: 3.96/5 interpretabi	○ (None mentioned)	○ (Not implemented)	3/5

	evaluation)	language + attention)	lity, 3.85/5 relevance)			
Heitz et al. (2024)	○ (No clinician input)	● (SHAP visualizations + stability metrics)	● (Feature stability assessment)	○ (None mentioned)	◐ (Real-time capability)	2/5
Ilias & Askounis (2022)	○ (No stakeholder input)	● (LIME explanations + natural language)	◐ (Statistical significance testing)	○ (None mentioned)	◐ (Interactive visualization proposed)	2/5
de Arriba-Pérez et al. (2024)	○ (No clinician input)	● (Component-based analysis + web interface)	◐ (Technical correlation analysis)	○ (None mentioned)	● (Real-time web application)	2/5
Ambrosini et al. (2024)	○ (No stakeholder input)	● (SHAP attribution + multi-language)	◐ (Cross-lingual validation)	○ (None mentioned)	◐ (Mobile app integration proposed)	2/5
Tang et al. (2023)	○ (No clinician input)	● (SHAP global/local + feature ranking)	● (ASR stability analysis)	○ (None mentioned)	◐ (Clinical decision support proposed)	2/5
Chandler et al. (2023)	○ (No stakeholder input)	● (Feature attribution + statistical testing)	● (Clinical correlation analysis)	○ (None mentioned)	● (Remote assessment tool)	2/5
Iqbal et al. (2024)	○ (No clinician input)	● (LIME + SHAP comparison)	◐ (Statistical validation)	○ (None mentioned)	◐ (Clinical screening tool proposed)	2/5

Legend: ● = Adequate; ◐ = Partial; ○ = Inadequate/Missing

Assessment Criteria:

- **Stakeholder Engagement**: Involvement of clinicians, patients, or other end-users in design/evaluation
- **Explanation Format**: Quality and appropriateness of explanation presentation (visual, textual, interactive)
- **XAI Evaluation**: Formal assessment of explanation quality, fidelity, or clinical utility
- **Training Materials**: Availability of user training, documentation, or support materials
- **Workflow Integration**: Consideration of how tool fits into existing clinical workflows

Key Findings: Only 2 studies (15%) achieved a clinical readiness score ≥3/5, with most studies (85%) scoring 2/5 or below. The primary gaps were lack of stakeholder engagement (92% inadequate) and absence of training materials (100% missing), highlighting critical barriers to clinical adoption despite technical advances in XAI methodologies.

PRISMA 2020 Checklist

Section and Topic	Item #	Checklist item	Location where item is reported
**TITLE**			
Title	1	Identify the report as a systematic review.	Title page
**ABSTRACT**			
Abstract	2	See the PRISMA 2020 for Abstracts checklist.	Abstract section
**INTRODUCTION**			
Rationale	3	Describe the rationale for the review in the context of existing knowledge.	Introduction, paragraphs 1-3
Objectives	4	Provide an explicit statement of the objective(s) or question(s) the review addresses.	Introduction, last paragraph
**METHODS**			
Eligibility criteria	5	Specify the inclusion and exclusion criteria for the review and how studies were grouped for the syntheses.	Methods: Eligibility criteria
Information sources	6	Specify all databases, registers, websites, organisations, reference lists and other sources searched or consulted to identify studies. Specify the date when each source was last searched or consulted.	Methods: Search strategy
Search strategy	7	Present the full search strategies for all databases, registers and websites, including any filters and limits used.	Methods: Search strategy
Selection process	8	Specify the methods used to decide whether a study met the inclusion criteria of the review, including how many reviewers screened each record and each report retrieved, whether they worked independently, and if applicable, details of automation tools used in the process.	Methods: Study selection
Data collection process	9	Specify the methods used to collect data from reports, including how many reviewers collected data from each report, whether they worked independently, any processes for obtaining or confirming data from study investigators, and if applicable, details of automation tools used in the process.	Methods: Study Selection and Data Extraction
Data items	10a	List and define all outcomes for which data were sought. Specify whether all results that were compatible with each outcome domain in each study were sought (e.g. for all measures, time points, analyses), and if not, the methods used to decide which results to collect.	Methods: Study Selection and Data Extraction, Appendix A
	10b	List and define all other variables for which data were sought (e.g. participant and intervention characteristics, funding sources). Describe any assumptions made about any missing or unclear information.	Methods: Study Selection and Data Extraction, Appendix A
Study risk of bias assessment	11	Specify the methods used to assess risk of bias in the included studies, including details of the tool(s) used, how many reviewers assessed each study and whether they worked independently, and if applicable, details of automation tools used in the process.	Methods: Risk of Bias Assessment
Effect measures	12	Specify for each outcome the effect measure(s) (e.g. risk ratio, mean difference) used in the synthesis or presentation of results.	Not applicable
Synthesis methods	13a	Describe the processes used to decide which studies were eligible for each synthesis (e.g. tabulating the study intervention characteristics and comparing against the planned groups for each synthesis (item #5)).	Methods: Data Synthesis and Risk of Bias Assessment
	13b	Describe any methods required to prepare the data for presentation or synthesis, such as handling of missing summary statistics, or data conversions.	Results: 3
	13c	Describe any methods used to tabulate or visually display results of individual studies and syntheses.	Results: 3

PRISMA 2020 Checklist

Section and Topic	Item #	Checklist item	Location where item is reported
	13d	Describe any methods used to synthesize results and provide a rationale for the choice(s). If meta-analysis was performed, describe the model(s), method(s) to identify the presence and extent of statistical heterogeneity, and software package(s) used.	Results:
	13e	Describe any methods used to explore possible causes of heterogeneity among study results (e.g. subgroup analysis, meta-regression).	Not applicable
	13f	Describe any sensitivity analyses conducted to assess robustness of the synthesized results.	Not applicable
Reporting bias assessment	14	Describe any methods used to assess risk of bias due to missing results in a synthesis (arising from reporting biases).	Not reported
Certainty assessment	15	Describe any methods used to assess certainty (or confidence) in the body of evidence for an outcome.	Results:
**RESULTS**			
Study selection	16a	Describe the results of the search and selection process, from the number of records identified in the search to the number of studies included in the review, ideally using a flow diagram.	Results
	16b	Cite studies that might appear to meet the inclusion criteria, but which were excluded, and explain why they were excluded.	Results
Study characteristics	17	Cite each included study and present its characteristics.	Results Tables 1-2
Risk of bias in studies	18	Present assessments of risk of bias for each included study.	Results Table 3
Results of individual studies	19	For all outcomes, present, for each study: (a) summary statistics for each group (where appropriate) and (b) an effect estimate and its precision (e.g. confidence/credible interval), ideally using structured tables or plots.	Results Tables 2
Results of syntheses	20a	For each synthesis, briefly summarise the characteristics and risk of bias among contributing studies.	Results Tables 3
	20b	Present results of all statistical syntheses conducted. If meta-analysis was done, present for each the summary estimate and its precision (e.g. confidence/credible interval) and measures of statistical heterogeneity. If comparing groups, describe the direction of the effect.	Not applicable
	20c	Present results of all investigations of possible causes of heterogeneity among study results.	Not applicable
	20d	Present results of all sensitivity analyses conducted to assess the robustness of the synthesized results.	Not applicable
Reporting biases	21	Present assessments of risk of bias due to missing results (arising from reporting biases) for each synthesis assessed.	Not reported
Certainty of evidence	22	Present assessments of certainty (or confidence) in the body of evidence for each outcome assessed.	Results
**DISCUSSION**			
Discussion	23a	Provide a general interpretation of the results in the context of other evidence.	Discussion, paragraphs 1
	23b	Discuss any limitations of the evidence included in the review.	Discussion, paragraphs 2-3
	23c	Discuss any limitations of the review processes used.	Discussion, paragraphs 4
	23d	Discuss implications of the results for practice, policy, and future research.	Discussion, paragraphs 4
**OTHER INFORMATION**			
Registration and protocol	24a	Provide registration information for the review, including register name and registration number, or state that the review was not registered.	Methods: The review protocol

PRISMA 2020 Checklist

Section and Topic	Item #	Checklist item	Location where item is reported
			was registered in PROSPERO (registration number: CRD42025637901)
	24b	Indicate where the review protocol can be accessed, or state that a protocol was not prepared.	Not reported
	24c	Describe and explain any amendments to information provided at registration or in the protocol.	Not reported
Support	25	Describe sources of financial or non-financial support for the review, and the role of the funders or sponsors in the review.	Funding section
Competing interests	26	Declare any competing interests of review authors.	Competing Interests section
Availability of data, code and other materials	27	Report which of the following are publicly available and where they can be found: template data collection forms; data extracted from included studies; data used for all analyses; analytic code; any other materials used in the review.	Not reported

From: Page MJ, McKenzie JE, Bossuyt PM, Boutron I, Hoffmann TC, Mulrow CD, et al. The PRISMA 2020 statement: an updated guideline for reporting systematic reviews. BMJ 2021;372:n71. doi: 10.1136/bmj.n71
